# Variable exon usage of differentially-expressed genes associated with resistance of sheep to *Teladorsagia circumcincta*

**DOI:** 10.1016/j.vetpar.2015.08.023

**Published:** 2015-09-15

**Authors:** Hazel Wilkie, Siyang Xu, Anton Gossner, John Hopkins

**Affiliations:** The Roslin Institute & R(D)SVS, University of Edinburgh, Easter Bush, Midlothian EH25 9RG, UK

**Keywords:** Sheep, *Teladorsagia*, Resistance, Cytokines, Gene variants

## Abstract

The resistance and susceptibility of sheep to the common abomasal nematode parasite, *Teladorsagia circumcincta* is strongly associated with the differential polarization of the immune response. Resistant animals control larval colonization by the production of a protective antibody response regulated by Th2 T cells. Susceptible sheep respond to infection by developing an inflammatory Th1/Th17 response that fails to control infection. Previous microarray analysis identified genes associated with T cell polarization that were differentially expressed between the resistant and susceptible sheep. RT-qPCR confirmed the microarray data for *ALOX15* and *IL13*. Both *ALOX15* exon 9 and *IL13* exon 4 were significantly increased in resistant animals and copy number RT-qPCR showed that expression levels of these exons were significantly negatively correlated with quantitative phenotypic traits, including abomasal worm counts and faecal egg counts. Sequencing of the intronic regions 5′ to these genes failed to identify any potential genetic links to differential exon usage.

## Highlights

• *ALOX15* and *IL13* genes show differential exon usage in relation to resistance of sheep to *Teladorsagia circumcincta*.
• Exon usage fully validated by relative RT-qPCR.
• *ALOX15* exon 9 and *IL13* exon 4 levels show significant negative correlation with adult worm count and faecal egg count and positive correlation with body weight and IgA antibody.

## Graphical abstract

**Figure:**
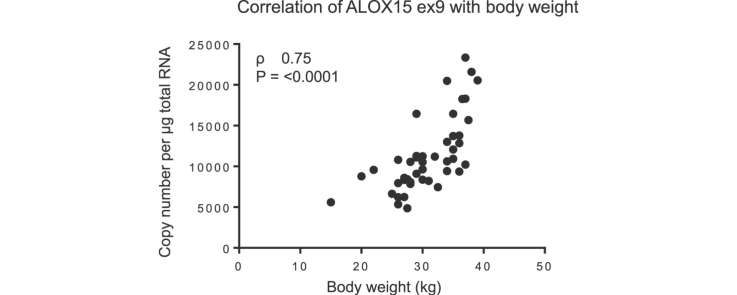

## 1 Introduction

The abomasal nematode, *Teladorsagia circumcincta* is one of the most common parasites of sheep in cool temperate regions (Urquhart et al., 1987) and is a major drain on the economics of sheep production (Nieuwhof and Bishop, 2005). Several studies have described genetic variation for parasite susceptibility within and between sheep breeds (Dominik, 2005, Mugambi et al., 1997, Terefe et al., 2007), and that faecal egg count (FEC) is a heritable characteristic (Davies et al., 2005) and can be used as a parameter for selective breeding for parasite resistance (Davies et al., 2005, Windon, 1990, Woolaston, 1997). Sheep selected for low FEC control infection by the acquisition of anti-parasite antibody (Stear et al., 1999) driven byTh2 cytokines (Craig et al., 2014), and there is a significant genetic relationship between IgA antibody levels and the ability to control infection (Stear et al., 1997, Strain et al., 2002). In contrast, sheep selected for high FEC produce little IgA antibody but instead generate an inflammatory Th1/Th17 response that fails to control infection and egg production (Gossner et al., 2012).

FEC and antibody levels have both been used as selectable markers of resistance (McRae et al., 2014, Sayers and Sweeney, 2005, Stear et al., 2006) but the identification of genetic markers has the potential for marker assisted selection (Davies et al., 2006). Both quantitative trait loci mapping and genome-wide association studies have been used to identify selectable genetic markers (Davies et al., 2005, Dominik, 2005, Riggio et al., 2013). More recently high throughput microarrays (Andronicos et al., 2010, Diez-Tascon et al., 2005) and digital gene expression (Pemberton et al., 2011) have been used to identify potential candidate genes for resistance to sheep gastrointestinal nematodes including *Haemonchus contortus* (Rowe et al., 2008) and *T. circumcincta* (Gossner et al., 2013, Knight et al., 2011). The aim of these studies was to identify genes that were differentially-expressed in pre-exposed (immune) vs. naïve or resistant vs. susceptible sheep and to try and explain the molecular basis of differential polarization of the immune response associated with each phenotype.

Microarray analysis of abomasal lymph nodes from infected sheep, selected on the basis of FEC, highlighted genes and physiological pathways related to the polarization of T cells and control of inflammation as being associated with resistance (Gossner et al., 2013). These data were obtained using the Affymetrix Ovine Gene 1.1 ST whole-genome array consisting of probes for all exons of each annotated transcript in the sheep Oar v2 genome assembly. The data were originally analysed at the gene-level, with exons summarized to genes, but this analysis also revealed that individual exons for some genes were not equally expressed.

Alternative splicing is a post-transcriptional mechanism for regulating gene expression; and within a gene the variable usage of (or part of) an exon or intron creates multiple distinct transcripts (Blencowe, 2006). Alternative splicing can modify coding sequence, but within the 5′ and 3′ UTRs it can also alter miRNA-binding and regulatory motifs and influence transcript expression levels or translation, and therefore can have a profound effect on the identity or expression levels of a protein (Martinez and Lynch, 2013).

The focus of this current study was to examine the differential usage of exons of selected genes in the resistant and susceptible sheep, as identified by microarray analysis (Gossner et al., 2013). The eight genes were chosen on the basis that they were differentially-expressed in resistant and susceptible sheep and have a role in T cell biology and inflammation and included arachidonate 15-lipoxygenase (*ALOX15*) and interleukin 13 (*IL13*), both of which were significantly increased in resistant animals and had variable exon usage. The microarray data were first validated by fold-change RT-qPCR for two exons of each selected gene; copy number RT-qPCR was then used to test the hypotheses that resistance/susceptibility to *T. circumcincta* is associated with differential exon usage within the selected genes and that individual exon usage correlates with selected quantitative parameters of resistance.

## 2 Materials and methods

### 2.1 Animals and tissues

Details of animals, infection protocols, phenotypic parameters and population genetic analyses have been described previously (Beraldi et al., 2008). Briefly, ∼3 months old Blackface female lambs originated from a flock used for quantitative trait analyses (Davies et al., 2006); all animals were housed in worm-free conditions. Ten sheep were sham infected controls and 45 sheep were infected experimentally with ∼2300 L3 *T. circumcincta* larvae three times a week for three months and sacrificed two days after the last infection. At post mortem ten of the infected group had no adult worms in the abomasal contents, while the other infected animals had adult worm count (AWC) within the abomasum at post mortem ranging from 80 to 11300 (Table S1). Animals were ranked (1–45) according to AWC and FEC (Beraldi et al., 2008). The relative concentration of serum IgA anti-*T. circumcincta* antibody was also measured for all these animals; and it was determined that both AWC and FEC were significantly negatively correlated with antibody levels and with body weight (BW) (Beraldi et al., 2008). The 7 most resistant (R) sheep (infection rank 1–7) had no detectable AWC or FEC, high IgA antibody levels and high body weight (BW). The 7 most susceptible (S) sheep (infection rank 39–45) were those with the highest AWC (mean 6000, maximum 11300), high FEC (mean 414, maximum 950), low IgA antibody levels and low body weight. Animal experiments were approved by University of Edinburgh Ethical Review Committee and conducted under an Animals (Scientific Procedures) Act 1986 Project Licence. Abomasal (gastric) lymph nodes (ALN) were removed at post mortem and stored at −80 °C in RNAlater (Ambion, Huntingdon, UK).

### 2.2 RNA extraction and cDNA synthesis

Total RNA was isolated from 0.02 g of tissue using the Ribopure Kit (Ambion, UK) according to the manufacturers’ instructions. Contaminating DNA was removed by On-column PureLink^®^ DNase I treatment (Ambion). The quantity, quality and integrity of the RNA samples were determined using a NanoDrop ND-1000 spectrophotometer (Labtech International Ltd.) and Agilent 2200 TapeStation system (Agilent Technologies); all had an RNA Integrity Number of >7.3. cDNA synthesis from 1 μg RNA was by SuperScript™ II Reverse Transcription Kit (Invitrogen, UK) using oligo-dT(_15_) primer and RNaseOUT (Invitrogen).

### 2.3 Cloning of ovine exons

The sequences of differentially-expressed probes of the Affymetrix Ovine Gene 1.1 ST Array (http://www.affymetrix.com/analysis/index.affx) were used to obtain full exon sequences using NCBI-BLAST against the sheep genome assembly, Oar v3.1 (http://www.livestockgenomics.csiro.au/sheep/oar3.1.php/). Exon-specific primers were selected using Primer-BLAST (http://www.ncbi.nlm.nih.gov/tools/primer-blast/) and reanalysed using Net Primer (www.premierbiosoft.com/netprimer/). Primers used for RT-PCR and RT-qPCR are shown in Table S2A. The location of the primers and their relationship to the locations of the Affymetrix Ovine Gene 1.1 ST whole-genome array probe sets is shown in Table S3. RT-PCR used the FastStart Taq DNA Polymerase Kit (Roche) following the manufacturer’s protocol. PCR products were analyzed by agarose gel electrophoresis, visualized by GelRed/UV transillumination, purified using MinElute PCR Purification Kit (Qiagen), ligated into pGEM-T Easy vector (Promega) and transformed into JM109High Efficiency Competent Cells (Promega). A random selection of color-screened clones were sequenced (Edinburgh Genomics; https://genomics.ed.ac.uk/) with SP6 and T7 primers using the BigDye^®^ Terminator v3.1Cycle Sequencing Kit (Applied Biosystems, UK).

### 2.4 Quantitative real-time PCR analysis

qPCR was performed using 1 μl template cDNA or linearized plasmid DNA (for the copy number analysis), 7.5 μl FastStart Universal SYBRgreen Master (Rox) 2× concentration (Roche), 0.1––0.3 μl primers and nuclease-free water to 15 μl final volume. All reactions were prepared using a CAS-1200™ Precision Liquid Handling System and performed on the Rotor-Gene Q (Qiagen). Cycle conditions were: 95 °C for 10 min and then 40 cycles of 95 °C for 10 s, annealing (Table S2A) for 15 s and 72 °C for 30 s followed by melt curve analysis. Optimised RT-qPCR primers had an efficiency between 95 and 105% and *R*^2^ of 0.98 and 0.99. Relative analysis was performed on the 7 most resistant sheep (ranked 1–7) and the 7 most susceptible sheep (ranked 39–45) (Table S1). Absolute expression (copy number) analysis was performed on all 45 infected animals. Transcripts were quantified in cDNA from three separate RT-qPCR reactions for each biological sample; each cDNA sample was assayed in triplicate with *GAPDH* housekeeping and no-template controls included in all runs.

Relative gene expression levels were calculated in GenEx 5.3.4.157 (MultiD Analyses AB, Sweden) using the comparative 2 − (ΔΔCq) method and normalized to the geometric mean of *GAPDH* and *SDHA*. Fold changes were calculated from ΔCq values using GenEx. To derive the copy number of the target sequence in all 45 sheep, a standard curve (linearized sheep *ALOX15* or *IL13* plasmid DNA) was used with a dynamic range that spanned at least five orders of magnitude. Copy numbers were calculated from Cq values using the following formula: molecules per μg = ((1 × 10^−6^)/(*M* g/mol)× [6.03 × 10^−23^ molecules/mol])

*M* = size of plasmid × 660 g/mol per bp. The expression levels were normalized by dividing the copy number derived from the standard curve by the calculated normalization factor for each individual sample.

### 2.5 Sequencing 5′ intronic regions

Genomic DNA (gDNA) was extracted from the ALN of 6 resistant (infection rank 1, 3, 4, 5, 6, 7) and 6 susceptible (infection rank 39, 41, 42, 43, 44, 45) sheep using the Wizard^®^ SV Genomic DNA Purification System (Promega). 10–20 mg of ALN tissue was incubated in the digestion solution at 55 °C with 300 rpm shaking overnight and then processed following the manufacturer’s protocol; 200 ng gDNA per PCR reaction was used as a template for cloning and sequencing of *ALOX15* and *IL13* promoter regions (primers and parameters Table S2B).

### 2.6 Statistical analysis

Relative gene expression levels were analyzed statistically in GenEx using an unpaired, 2-tailed *t*-test to determine the difference between groups. *ALOX15* and *IL13* copy number results were analyzed in GraphPad Prism v 5 (Graph Pad Software, USA). The data were grouped into resistant, susceptible and intermediate (*n *= 15 per group) and a Kruskal–Wallis test was performed to determine overall significance, with Dunn’s multiple comparison test within the Kruskal–Wallis to determine significance between groups. The correlations between transcript levels and quantitative phenotypes were analyzed with Spearman’s rank correlation coefficient (*r*_s_). *p*-values less than 0.05 were considered statistically significant.

## 3 Results

### 3.1 RT-qPCR validation of array analysis

Transcriptome analysis of the ALN has been described previously (Gossner et al., 2013). In brief, cDNA from the abomasal lymph node of 7 resistant, 7 susceptible and 7 uninfected sheep was hybridized to Affymetrix Ovine Gene 1.1 ST Array and analysed, with exons summarized to genes, using the mean expression of all the exons of a gene. These data and protocols are available at ArrayExpress accession number E-MTAB-1580. In this current study the same dataset was used but without summarizing to genes. Of the 165740 exons interrogated, 1196 were significantly differentially-expressed within their gene in the R vs. S comparison, with a fold change ≥2 and *p*-value ≤0.05 (one-way between-subject ANOVA, unpaired); 930 exons were increased and 266 exons were repressed. Eight genes were selected, based on each being significantly differentially-expressed in the R vs. S comparison, their role in T cell biology and inflammation as well as significant differential exon usage within the gene (Table S4). RT-qPCR was developed for two exons of each gene including one with significant differential-expression.

Comparison of the array and RT-qPCR fold change analysis for the exons of the selected genes is shown in Table 1. Significant differential exon expression was validated, by RT-qPCR, only for *ALOX15* and *IL13*. Exons 9 and 14 of *ALOX15* were 22.02 and 7.44 fold increased respectively, in the resistant compared to susceptible sheep as assessed by array, and 5.3 (*p* = 0.001) and 1.5 fold (*p* = 0.03) by RT-qPCR. Exons 1 and 4 of *IL13* were 3.6 and 6.91 fold increased by array and 3.77 (*p* = 0.0002) and 5.16 fold (*p* = 5.2*E* − 6) by RT-qPCR. When the data were summarized to the whole gene, *ALOX15* was 5.81 fold higher, and *IL13* was 3.17 fold higher in the resistant sheep (Gossner et al., 2013). Differential exon expression of the other six genes was not confirmed by RT-qPCR, although all but *CD109* demonstrated significant differential expression for at least one exon in the R vs. S comparison.

### 3.2 Association of exon copy number and quantitative phenotype

*ALOX15* and *IL13* were chosen for copy number measurement in the ALN in all 45 infected animals. The expression levels of *ALOX15* exon 9 was significantly higher than exon 14 (mean 11313 ± 4547 and 7729 ± 2360 copies per μg total RNA respectively, *p* = 0.0003). Similarly IL13 exon 4 was expressed significantly higher that exon 1 (152.377 ± 81.222 and 72.406 ± 43.858, *p* < 0.0001). The data were further analysed by dividing the 45 animals into three groups; the fifteen most resistant sheep with mean AWC of 59 and mean FEC of 1.67; a susceptible group of fifteen sheep with mean AWC of 5167 and mean FEC of 288; and the fifteen intermediate sheep with mean AWC of 1508 and mean FEC of 82 (Table S1).

**Fig. 1:**
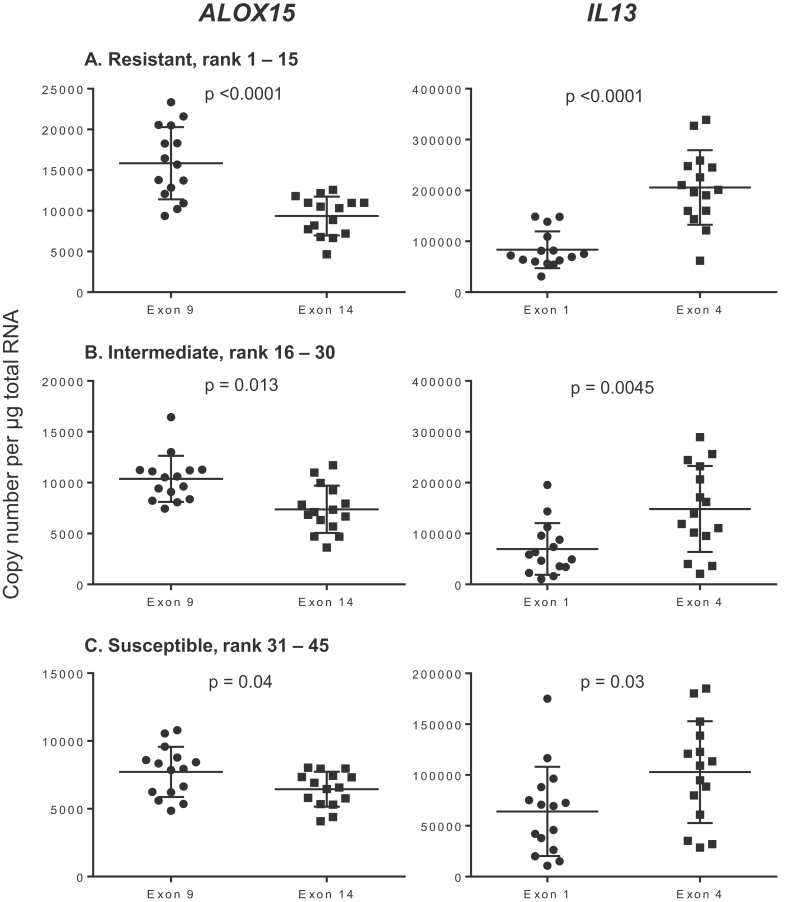
Expression of *ALOX15* exons 9 and 14, and *IL13* exons 1 and 4 in the ALN of *T. circumcincta* infected sheep. Copy number per μg total RNA. A, rank 1–15 resistant sheep (FEC < 10). B, rank 16–30, intermediate (FEC 10–219). C, rank 31–45 susceptible (FEC > 220). Error bars are means ± SD.

Quantification of *ALOX15* (Fig. 1) showed that exons 9 and 14 expression levels were significantly different in the resistant, rank 1–15 (15.842 ± 4434 for exon 9 and 9360 ± 2382 copies per μg RNA for exon 14, *p* < 0.0001), intermediate, rank 16–32 (10377 ± 2272 and 7381 ± 2330, *p* = 0.013) and susceptible groups, rank 31–45 (7719 ± 1846 and 6446 ± 1286, *p* = 0.04). Measurement of the *IL13* exons also showed significant differential expression of exon 1 and exon 4 in all three groups; 83418 ± 36148 for exon 1 and 205953 ± 73295 for exon 4, *p* < 0.0001 in the resistant group; 69660 ± 50.941 and 148318 ± 84464, *p* = 0.0045 in the intermediate group, and 64139 ± 43.959 and 102858 ± 50067, *p* = 0.03 in the susceptible group.

**Fig. 2:**
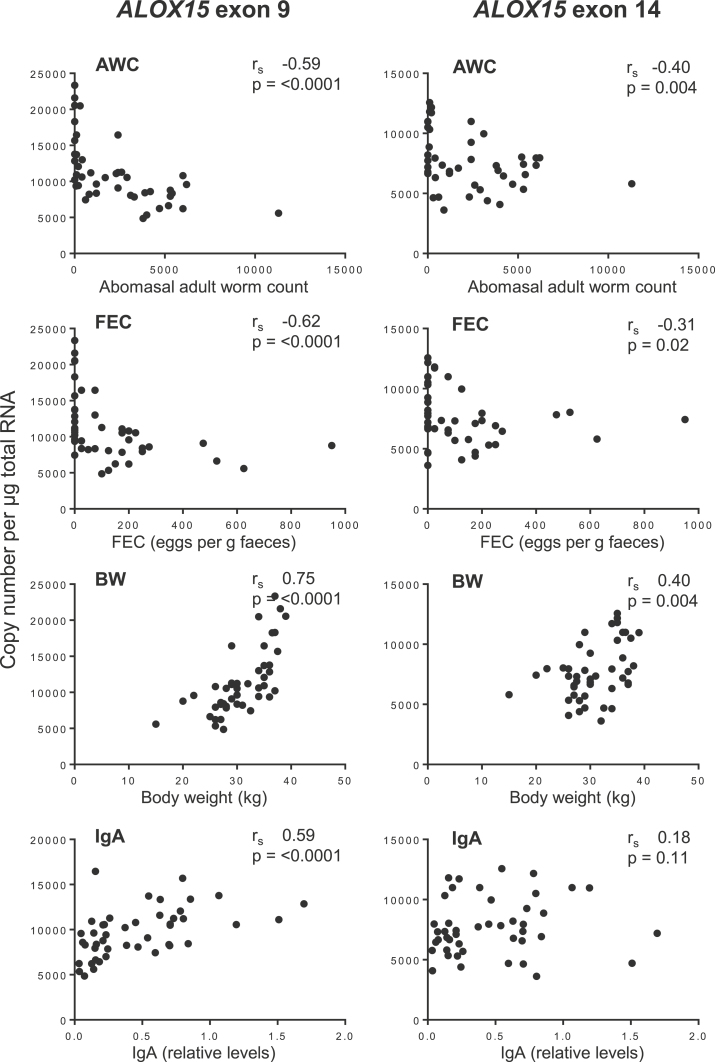
Correlation analysis of the phenotypic parameters, AWC, FEC, BW and IgA with *ALOX15* exon 9 and 14 copy number per μg total RNA in ALN of *T. circumcincta* infected sheep. *r*_s_—Spearman’s rank correlation coefficient.

Spearman’s rank correlation analysis was used to quantify the correlation of expression levels of each exon in relation to the quantitative phenotypes, FEC, AWC, BW and IgA antibody levels (Table S1). *ALOX15* exon 9 was significantly negatively correlated with AWC (*r*_s_ −0.59, *p* < 0.0001) and FEC (*r*_s_ −0.62, *p* < 0.0001), and significantly positively correlated with BW (*r*_s_ 0.75, *p* < 0.0001) and IgA (*r*_s_ 0.59, *p* < 0.0001), exon 14 was also significantly negatively correlated with AWC (*r*_s_ −0.40, *p* = 0.004) and FEC (*r*_s_ −0.31, *p* = 0.02), and significantly positively correlated with BW (*r*_s_ 0.40, *p* < 0.004), but not with IgA (*r*_s_ 0.18, *p* = 0.11) (Fig. 2).

**Fig. 3:**
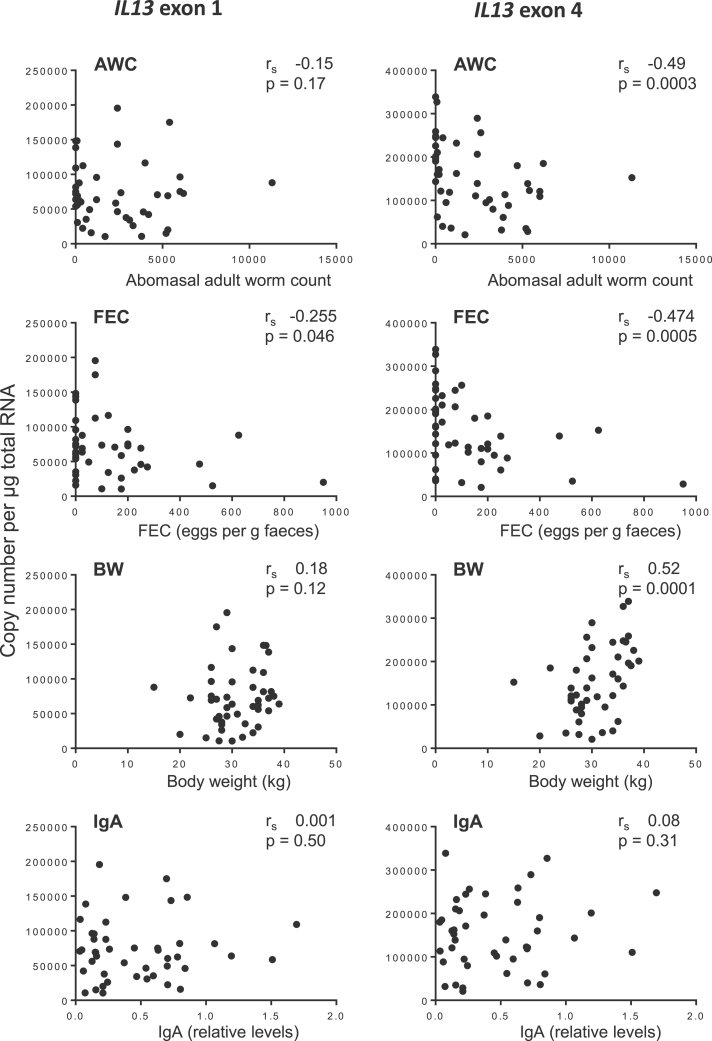
Correlation analysis of the phenotypic parameters, AWC, FEC, BW and IgA with *IL13* exon 1 and 4 copy number per μg total RNA in ALN of *T. circumcincta* infected sheep. *r*_s_—Spearman’s rank correlation coefficient.

Similar analysis for *IL13* (Fig. 3) showed that exon 4 expression was significantly negatively correlated with AWC (*r*_s_ −0.49, *p* = 0.0003) and FEC (*r*_s_ −0.474, *p* = 0.0005) and positively correlated with BW (*r*_s_ 0.52, p = 0.0001) but not with IgA levels (*r*_s_ 0.08, *p* = 0.31). *IL13* exon 1 expression was also significantly negatively correlated with FEC (*r*_s_ −0.255, *p* = 0.046) but there were no significant correlations between exon 1 and AWC (*r*_s_ −0.15, *p* = 0.17), BW (*r*_s_ 0.18, *p* = 0.12) or IgA (*r*_s_ 0.001, *p* = 0.50).

### 3.3 Sequence of 5′ intronic or UTR regions of ALOX15 and IL13

Polymorphisms in intronic promoter or 5′ UTR regulatory elements (Black, 2003), possibly associated with differential exon usage, were examined by sequencing approximately 1000 bp 5′ to the translation start site of both *ALOX15* (LN864492) and *IL13* (LN864491) from 6 R and 6 S sheep. Ten single base variations were identified in the *ALOX15* region (g.66T>C; g.227C>G; g.246C>G; g.606T>C; g.654G>A; g.656G>C; g.697T>C; g.836T>C; g.960G>A; g.1036A>G) but none segregated with phenotype. An initiator codon variant (A/G) was also identified in two animals, which has already been identified in other sheep breeds (NCBI dbSNP; rs422045752). The only variation found with *IL13* was the insertion of three nucleotides (g.674_675insGAA) 222 nucleotides 5′ to the translation start site in one animal (lamb 110, rank 7), which was heterozygous at this locus.

## 4 Discussion

The resistance of sheep to the gastrointestinal nematode parasite *T. circumcincta* has a major host genetic component (Murphy et al., 2010, Riggio et al., 2013) and differential susceptibility to this parasite is clearly associated with T cell activation and inflammation (Gossner et al., 2013, Gossner et al., 2012). In addition, mouse models of nematode infections show that variations of genes associated with Th1, Th2 and Th17 T cell maturation are involved in the genetics of resistance (Filbey et al., 2014, Finkelman et al., 1997). Most studies in sheep have focussed on the important role for transcription regulation and primary sequence differences of these genes (Gossner et al., 2013, Riggio et al., 2013); however this current study concentrates on differential exon usage as a source of gene variants. Alternative splicing is a significant cause of protein variation (Martinez and Lynch, 2013) especially in genes expressed in the immune system (Lynch, 2004) including cytokines and receptors involved in T cell polarization (Gaudreau et al., 2012, Martinez et al., 2012); and the original selection of the eight genes was made on the basis that they were differentially-expressed in resistant and susceptible sheep (Gossner et al., 2013) as well as being involved in T cell biology and inflammation.

Fold-change RT-qPCR resulted in the validation of the microarray data of two of the eight selected genes, *ALOX15* and *IL13*. IL13 plays a major role in resistance to gastrointestinal nematodes. In *T. circumcincta* infected sheep it was the most significant up-regulated gene in the R vs. S comparison by both microarray and RT-qPCR array (Gossner et al., 2013). It is also the dominant cytokine in the control of murine *Nippostrongylus brasiliensis* infection (Mckenzie et al., 1998) and seems to be equally involved with IL4 in the development of protective immunity to *Trichuris muris* and *Heligomosoides polygyrus* (Bancroft et al., 1998). IL13 is largely a product of antigen-activated Th2 cells but is also synthesized by mast cells and eosinophils (Gessner et al., 2005). IL13 shares many biological functions with IL4, including the regulation of Th2 development and consequently antibody production and heavy chain switch; it also inhibits inflammatory cytokine production and promotes mast cell proliferation (Minty et al., 1993) and tissue remodelling after parasite-induced injury (Wynn, 2003). *IL13* is encoded by four exons on the minus strand of ovine chromosome 5 (NC_019462.1); exon 4 encodes 108 bp at the 3′ end of the coding region (19.262.871–19262764) and the 862 bp 3′ UTR (19.262.763–19261902). The consistent differential expression of both exons between the R and S groups, and the highly significant correlation of exon 4 levels and sheep rank phenotypes (AWC and FEC) as well as BW, argues against a simple technical explanation for differential exon usage. Expression levels of exon 4 are likely to represent full length and functional *IL13* transcripts, which may explain why quantitative levels of *IL13* exon 4 are significantly positively correlated BW and consequently negatively correlated with AWC and FEC. The lack of correlation between *IL13* exon 4 and IgA (*r*_2_ 0.08, *p* = 0.31) may be due to the fact that the *IL13* levels are transcripts at a single time point (12 weeks post-infection), but that serum IgA had accumulated over time.

Another major function of IL13 (and IL4) is the promotion of *ALOX15* expression (Nassar et al., 1994) and the consistent up-regulation of all the *ALOX15* exons in resistant sheep could be explained by the high levels of these cytokines in those sheep. ALOX15 is one of a range of at least six isoenzymes that oxygenate arachidonic acid leading to the production of the lipoxins (Samuelsson et al., 1987). These function to inhibit leukotrienes and therefore are broadly anti-inflammatory. In addition ALOX15 stimulates the clearance of apoptotic cells and promotes tissue healing (McMahon et al., 2001), which is seen in resistant sheep (Gossner et al., 2012). *ALOX15* has not been annotated in the Oar v3.1 genome assembly but analysis using the *Bos taurus ALOX15* sequence (NM_174501.2) identifies that the sheep gene is encoded by 14 exons on the plus strand of chromosome 11 (NC_019468.1). Exon 9 encodes 90 bp within the coding region (1247–1337 from the translation start site, 26326237–26326327) and exon 14 encodes the terminal 180 bp of the coding region and the complete 3′ UTR (26327916–26328935). As with *IL13*, it is likely that the *ALOX15* transcript that includes exon 9 encodes the full length and functional enzyme, and therefore there is a highly significant relationship between exon 9 expression levels and phenotypic parameters of parasite resistance. One transcript and three predicted transcript variants of *ALOX15* have been described in cattle, with the X2 variant (XM_005220190.2) having a deleted exon 7. However, no functional consequences of these variants have been described.

Control of exon usage and differential splicing can be associated with intronic regulatory elements (Black, 2003), often in the proximal 1000 bp 5′ to the translation start site. These regions were sequenced for both *ALOX15* and *IL13* from 6 resistant (rank 1–6) and 6 susceptible (rank 40–45) sheep; and although variants were identified for both genes, none segregated with resistance and susceptibility phenotype.

In conclusion, we have validated differential exon usage of two genes that were expressed in the ALN of sheep showing differential resistance to the abomasal nematode *T. circumcincta*. Both *ALOX15* exon 9 and *IL13* exon 4 were significantly increased in resistant animals and expression levels of these exons were negatively correlated with quantitative phenotypic traits, including AWC and FEC. Consequently, they represent potential markers for selection for resistance to a common and economically important gastrointestinal parasite.

## Conflict of interests

The authors declare that they have no conflicts of interest.

## Figures and Tables

**Table 1:** Differentially-expressed genes in R vs. S comparison with differential exon usage.

Gene symbol	Gene name	Exon	FC^a^	*p* value	FC^a^	*p* value^b^
			Microarray		RT-qPCR	
*ALOX15*	Arachidonate 15-lipoxygenase	9	22.03	0.003	5.3^c^	0.001
14	7.44	0.001	1.5	0.03
*CD109*	CD109 molecule	11	6.36	0.01	−1.0	0.09
19	1	0.05	−1.1	0.08
*CD163*	CD163 molecule	1	1	0.05	−2.4	0.01
9	−5.42	0.02	−1.6	0.04
*CPA3*	Carboxypeptidase A3 (mast cell)	3	8.0	0.004	2.0	0.02
5	2.2	0.03	1.5	0.04
*EMR3*	egf-like module containing, mucin-like, hormone receptor like-3	9	6.83	0.008	2.2	0.06
11	3.58	0.01	2.0	0.01
*IL13*	Interleukin 13	1	3.6	0.0001	3.77^c^	0.0002
4	6.91	0.0002	5.16	5.2*E* − 6
*KIT*	v-Kit Hardy–Zuckerman 4 feline sarcoma viral oncogene homolog	16	6.16	0.006	1.1	0.08
21	2.06	0.01	1.2	0.05
*MAP3K5*	Mitogen-activated protein kinase kinase kinase	26	1	0.05	−1.5	0.02
28	7.73	0.01	−1.3	0.03

a Fold change R vs S comparison.

b *p* value R vs S.

c *p* ≤ 0.05 in the comparison of the two exons.
