# Supplementary material for: Variable exon usage of differentially-expressed genes associated with resistance of sheep to Teladorsagia circumcincta
Source: Vet Parasitol. 2015 Sep 15;212(3-4):206–13. doi: 10.1016/j.vetpar.2015.08.023 (PMC4608359; doi:10.1016/j.vetpar.2015.08.023)
Supplement: Supplementary file 1 [file mmc1.docx]

**(A) ALOX15 5’ sequence**

sheep ---------------------------------------------------------TGC

cattle GAGAGCTCGGAGGCCGCTGGGTAAAGCGCTTTAGTGCGGGACGCGGCACACAGCAAGTGC

***

sheep TCAATAAGTGTTCTTGCTGCCCGGCCTGCTTGGAATTGGGATAGTCTCGTGGTT------

cattle TCAATAAGTGTTCT-TCTGCCCGGCCTGCTTGGAATTGGGATAGTCCCGTGGTTCTTCAG

************** ****************************** *******

sheep --CTCGGTCT**T**TTCCGGAAACTCCGGGGTCCTTGGAGCGGACATCGGTCTCCTGTTTCCT

cattle AACTCGGTCTTTTCCGGAAACTCCGGGGTCCTTGGAGCGGACATCGGTCTCCTGTTTCCT

**********************************************************

sheep CTTCTTAGAAGCTGCTATTTCAAATCTTCTGTAAGGTTGCTAGATCTCCGACCTTGCGGG

cattle CTTCTTAGAAGTTGCTATTTCAGATCTTCTGTAAGGTTGCTAGATCTCCGACCTTGCAGG

*********** **********.**********************************.**

sheep CTTTATCTCCGGGATGGAGCGGGAATGGGGACGCGGGAAGCGGGGGAGAAC**C**GAAGAAGC

cattle TTTTATCTCCGGGATGGAGCGAGAATGGGGACGCGGGAAGCGGGGGGGAACGGAAGAAGC

********************.************************.**** ********

sheep CGCCTGGTGA**C**AGCACTCTTATTTCCCGGCCCACCCTGTTTCCCATCACGTCTACCTGGC

cattle CGCCTGA---GAGCACTCTTATTTCCCGGCCCACCCTCTTTCCCATCACATGTACCTGGT

******. ************************** ***********.* *******

sheep ATTCGCCCCTGGCTCCTGTTACTTTTTGCCTCCGCCGAGTCCCAGATTTCCATCTCCCAT

cattle ATTCGCCCC-------TGTTACGTTTTGCCTCCGCCGATTCCCAGATTTCCGTCTCCCAC

********* ****** *************** ************.*******

sheep CCGGCTCTCCCCAAGAGCCCCAGACCCGACGTTTATCCCACGGACGCTTCAGAGTACCGT

cattle CCGGCTCTCCCCAAGACCCCCAGACCCGACGTTTATCCCATGGACGCTTCAGAGAACCGT

**************** *********************** *************:*****

sheep GCTCACGTGAGGTCCTCCCCTCTGCCCACCTCCCAAACCGGTCCTCTTTTCTCCCAGGTG

cattle GCTCACGTGAGGTCCTCCCCTCCTCCCACCTCCCAAACCGGTCCTCTTTTCTCCCAGGTG

********************** ************************************

sheep CACTCGCGCCTCCGCCTCTTTTCGCCTGCGTCTAACCAAAAACCCGGTGTAGTCCAGTTC

cattle CACTCGCGCCTCCGCCTCTTTTCGCCTGCGTCTAACCAAAAATCCAGCGTAGTCCAGTTC

****************************************** **.* ************

sheep ATCTCTTAGACATTTCTCGCACCCTTGCCCTGATGTCCATCCCACTCTGACGACCAGTGT

cattle ATCTCTTAGACATTTCTCGAATCCGTGCCCTGATGTCCATCCCACTCTGACGGCCAATGT

*******************.* ** ***************************.***.***

sheep CCCCTCGAGC**T**TACTACATCACCTTCTTCCTCTTCGCCCGCCTCATTGCTCCAGCTCC**G**A

cattle CCCCTCGAGCCTACTACATCACCTTCTTCCTCTTCGCCCGCCTCATTGCTCCAGCTCCAA

********** ***********************************************.*

sheep **G**CCCTTCTGCAGCCAGCGATTTCTTCCCAATACATTCCCAC**T**CTTAAAACATCCCCGCAG

cattle GCCCTTCTGCAGTCAGCGATTTCTTCCCAATACATTCCCACCCTTAAAACATGCCCCCAG

************ **************************** ********** *** ***

sheep GGCTTCCCTGGGGGCCCAGTGGTAAAGAATCCACCTGCTAATGCAGGAGCCTTGGCTTCC

cattle GGCTTCCCTGGGGGACCAGTGGTAAAGAATCCACCTGCTAATGCAGGAGCTTTGGCTTCC

**************.*********************************** *********

sheep ATCCTTGGTCTGGGAAGATCCCACATGCCTCCGAGCAACTAAGGCCCTGCGCCACATTTA

cattle ATCCTTGGTCTGGGAAGATCCCACATGCCTCCGAGCAACTAAGGCCCTGTGCCACATCTA

************************************************* ******* **

sheep **T**GGAGTCTGTGCTCCAGAGCCGGGGAGCCACAAGTGCTGAGCCCACGGCCCTGGTGACAC

cattle TGGAGTCTGTGCTCCAGAGCCGGAGAGCCACAAGTGCTGAGCCCACGGCCCTGGTGACAG

***********************.***********************************

sheep GGTAGGGGGCCGCGGTAACTGTCTGGTCACCAGTGAGCTCGCCCAGGGTGCAGCCCAGTG

cattle GGTAGGGGGACGCGGTAACTGTCTGGTCACCAGTGAGCTCGCCCAGGGCGCAGCCCAGTG

*********.************************************** ***********

sheep CTCC**G**CACAATCAGGGACTTCGGGGAGTCACGCCCCGCTCCATCTTCTGTCCCCCACCTC

cattle CTCCACACAATCAGGGACTTCGGGGAGTCATGCCCCTCTCCATCTTTTGTCCCCCACCTC

****.************************* ***** ********* *************

sheep ACCTGGGGCTGAGGAATTCTATGACCGAGGACGGGGTCGTGGAGCCAACTGCTTGGTGCG

cattle ACCCGGGGCTGAGGAATTCTATAACCGAGAATGGGGTCGTGGAGCCAACTGCTTGGTGCT

*** ******************.******.* ***************************

sheep TGA---------------------------------------------------------

cattle CGCGCCCGCTTCCTCTCTGGCTCTTTTTGCGCCGCTTTGCCAGAGGCCCAGAGGGAAGGG

*.

polymorphisms (Bold)

g.66T>C; g.227C>G; g.246C>G; g.606T>C; g.654G>A; g.656G>C; g.697T>C; g.836T>C; g.960G>A; g.1036A>G.

Fig. S1. (A) Sequence of intron immediately 5’ of sheep ALOX15. Comparison of sheep (LN864492) and cattle (XM_005220190.2) sequences.

Box; translation start site. Underline; predicted 5’ UTR (Neural Network Promoter Prediction <http://www.fruitfly.org/seq_tools/promoter.html>). Bold; polymorphic sites.

**(B) IL13 5’ sequence**

sheep CCTGCAAGGATCCCCGAATCTGACTGGATCTTTCAATGGAGACCTGGGAATCTGAAATTC

cattle -------GGATCCCTGAATCTGACTGGATCTTTCAATGGAGACCTGGGAATCTGAAATTC

******* *********************************************

sheep CACGGTCCTTCCCACACTGGGGCAACAGGGAACCAAATGGGTTAAAAGAAATGAACATCA

cattle CACAGTCCTTCCCACACTGGGGCAACAGGGAACCAGATGGGTTAAAAGACATGAACATCA

***.*******************************.*************.**********

sheep CTCT---TTTATCCTGTTTGAATTTTGTGTTCAGAGCAAGCATTGTTTTGGTAATGTAAC

cattle CTCTTCTTTTATCCTGTTTGAATTTTGTATCCAGAGCAAGCATTGTTTTGGTAATGTAAC

**** *********************.* *****************************

sheep AGAACTGTTCAGTGAAAAAACAATCCCTCCAGATTCCCACATCTGATAGACTGTGATCAG

cattle AGAACTGTTCAATGAAAAAATAATCCCTCCAGATTCCCACATCTGATAGACTGTGATCAG

***********.******** ***************************************

sheep AGCACTGTCTCTCCAGCATTTCTGGTGGCCCAAAGGGATCTGCGGACAGGAGCTGGGTGG

cattle AGCACTGTCTCTCCAGCATTTCTGGTGACCCAAAGGGATCTGCGGACAGGAGCTGGGTGA

***************************.*******************************.

sheep GCACTCGCTAAAAGTGCTGGAGTTCCAGCTGGCATAGTTGGCATCAGACTGCCCTCGGGG

cattle GCACTCGCTAAAAGTGCTGGAGTTCCAGCTGGCATAGTTGACATCGGGCTGCCCTCGGGG

****************************************.****.*.************

sheep TCCTCTCGTTCACCCCTGCAGTAAGTTTATCCAGATCTCGAAACTGCCCTAGACCCTTCT

cattle TCCTCTCATTCACCCCTGCAGTAAGTTCATCCAGATCTCGAAACTGCCCTAGACCCTTCT

*******.******************* ********************************

sheep CAGTAATAAGTCCACAA**---**ATCAAATTCTTTCCTTTATGTGACACTGGATTTTCCACAA

cattle CAGTAATAAGTCCACAAGAAATCAAATTCTTTCCTTTATGCGACACTGGATTTTCCACAA

**************************************** *******************

AP1 AP3 Gata3

sheep AGTAAAATTAAGATGAGTAAAGATGTGGTTTGTAGATAGTGCCCAACAAAGCGGAGACCA

cattle AGTAAAATTAAGATGAGTAAAGATGTGGTTTGTAGATAGTGCCCGACAAAGCAGAGACCA

********************************************.*******.*******

TATA box

sheep GGGTGTGAGGCGTCACCACTTGGGCC**TATAAA**AGCTGCCACAAGCGCCTAAGGCCACAAG

cattle GGGTGTGAGGCGTCACCACTTGGGCCTATAAAAGCTGCCACAAGTGCCTAAGGCCACAAG

******************************************** ***************

sheep ACGCGCAGCTTAGGCCAGCCTATGCGTCTGCTTCTCAGTT--CTTCTGTGTTGTTCTAGG

cattle ACACGCAGCTTAGGCCAGCCTATGCGTCTGCTCCTCAATTTCTTCCTGTGCTGTTCTAGG

**.***************************** ****.** * ***** *********

sheep CTCCATGGCGCTCTTCTTGACCGTGGTCGTTGTTCTTACCTGCTTTGGTGGCCTCGCCTC

cattle CTCCATGGCGCTCTTATTGACCGCGGTCATTGTTCTTATCTGCTTTGGTGGCCTCACCTC

***************.******* ****.********* ****************.****

Fig. S1. (B) Sequence of intron immediately 5’ of sheep *IL13*. Comparison of sheep (LN864491) and cattle (AJ132441_1) sequences.

Box; translation start site. Underline; Exon 1 5’ UTR. Bold underline; TATA box. Double underline; promoter binding sites.
