# Supplementary material for: Variable exon usage of differentially-expressed genes associated with resistance of sheep to Teladorsagia circumcincta
Source: Vet Parasitol. 2015 Sep 15;212(3-4):206–13. doi: 10.1016/j.vetpar.2015.08.023 (PMC4608359; doi:10.1016/j.vetpar.2015.08.023)
Supplement: Supplementary file 3 [file mmc3.docx]

Table S2.

(A) Sequences and PCR conditions of exon-specific primers

| Gene | Exon | Primer  sequences | Product size (nt) | Anneal.  Temp | PCR efficiency | R^2^ |
| --- | --- | --- | --- | --- | --- | --- |
| ALOX15 | 9f | TCCACACCTGCGATACACCA | 73 | 61^o^C | 1.03 | 0.97 |
|  | 9r | GAAGACTCCCTTGTCAGCGA |  |  |  |  |
|  | 14f | GAAGTTCAGGGAGGAGCTGG | 82 | 62^o^C | 1.00 | 0.97 |
|  | 14r | CAGGTATTCGTAGGGCCAGT |  |  |  |  |
|  |  |  |  |  |  |  |
| CD109 | 19f | ACTGTGTCCTCAGATGCCATT | 82 | 60^o^C | 0.97 | 0.98 |
|  | 19r | CTAAGTAGAGGTTGAGTTTCCCAT |  |  |  |  |
|  | 11f | AAGGCCCTATCTGAATTTGCAGC | 135 | 62^o^C | 1.00 | 0.95 |
|  | 19r | TGACATCTGGAGGAGAAAGCG |  |  |  |  |
|  |  |  |  |  |  |  |
| CD163 | 1f | CTCAAAGGTGGAGGTAGCCG | 268 | 60^o^C | 0.97 | 0.98 |
|  | 1r | GTTCACAACTAAGCCCACCC |  |  |  |  |
|  | 9f | CGGAGTCAGACACTATATCCATGC | 89 | 61^o^C | 0.97 | 0.97 |
|  | 9r | CCTATGCAAGGAACACCATTTTCT |  |  |  |  |
|  |  |  |  |  |  |  |
| CPA3 | 3f | GCTTACAACCTGGGCATCAAAC | 134 | 61^o^C | 0.95 | 0.99 |
|  | 3r | TTGACAATAAATTTGACAGCAGGC |  |  |  |  |
|  | 5f | AACACCAACGATCCATGTCAG | 192 | 61^o^C | 0.95 | 0.97 |
|  | 5r | GTTCTTGTGGTTTGGTGGCAG |  |  |  |  |
|  |  |  |  |  |  |  |
| EMR3 | 9f | CGCAGGTGCCTTACACTATC | 129 | 61^o^C | 0.95 | 0.98 |
|  | 9r | CTGCAGAAATGGCCACGAT |  |  |  |  |
|  | 11f | TCTGCGTCTACTGGAAAGGC | 132 | 61^o^C | 1.02 | 0.98 |
|  | 11r | AGGCCATGAGAACAGCGAAA |  |  |  |  |
|  |  |  |  |  |  |  |
| IL13 | 1f | CTCCATGGCGCTCTTCTTGA | 82 | 62^o^C | 1.05 | 0.99 |
|  | 1f | AGAGGAAGGCACAGGGTTTG |  |  |  |  |
|  | 4f | GCCCAGGCACATTCCTTCTT | 95 | 61^o^C | 0.95 | 0.99 |
|  | 4r | GGCAGTAACAGTCCCTCCTAAC |  |  |  |  |
|  |  |  |  |  |  |  |
| KIT | 16f | AAGAGACGTGACTCCTGCTA | 115 | 61^o^C | 0.95 | 0.95 |
|  | 16r | ATTCTTTGAGGCAAGGAACGC |  |  |  |  |
|  | 21f | GCAGGGTTTGTGTGTTGTCTC | 94 | 61^o^C | 0.95 | 0.95 |
|  | 21r | GCCACAGTTCTCTAAATGAAAGCA |  |  |  |  |
|  |  |  |  |  |  |  |
| MAP3K5 | 26f | CACAGTGCTCCACCTTCTCC | 90 | 61^o^C | 0.97 | 0.97 |
|  | 26r | AATCCTGTGAAGCGTAGCCC |  |  |  |  |
|  | 28f | CGATGCCTTCCGACAACCAT | 112 | 61^o^C | 1.00 | 0.98 |
|  | 28r | TGACTGCAGAGAGTCTGAATTAGT |  |  |  |  |

(B) Sequences and PCR conditions of genomic promoter primers

| Gene | Access No. | Primer sequence | Size | PCR parameters |
| --- | --- | --- | --- | --- |
| *ALOX15* |  | F: GCTCAATAAGTGTTCTTGCTGCC  R: CACGCACCAAGCAGTTGG | 1076bp | 95 ^o^C 4min,  (95 ^o^C 15s, 62 ^o^C 20s, 72 ^o^C 70s) x35, 72 ^o^C 7min |
| *IL13* |  | F: ACGACTCAGAGAGCACAGGATG  R: AGAGGAAGGCACAGGGTTTG | 974bp | 95 ^o^C 4min,  (95 ^o^C 15s, 62 ^o^C 20s, 72 ^o^C 60s) x35, 72 ^o^C 7min |
