# Supplementary material for: Variable exon usage of differentially-expressed genes associated with resistance of sheep to Teladorsagia circumcincta
Source: Vet Parasitol. 2015 Sep 15;212(3-4):206–13. doi: 10.1016/j.vetpar.2015.08.023 (PMC4608359; doi:10.1016/j.vetpar.2015.08.023)
Supplement: Supplementary file 4 [file mmc4.docx]

| Table S3.  Primer and probe set locations on mRNA and OARv3.1 genomic sequences of *ALOX15* and *IL13* | | | | | |
| --- | --- | --- | --- | --- | --- |
| ***ALOX15*** |  | | NM_174501.2^1^ | NC_019468.1^3^ |  |
| Exon 9 |  | | 1168 - 1258 | 26326237 - 26326327 |  |
| 9f | TCCACACCTGCGATACACCA | | 1178 - 1197 | 26326247 - 26326266 |  |
| 9r | GAAGACTCCCTTGTCAGCGA | | 1231 - 1249 | 26326300 - 26326319 |  |
| probe set 14724110 |  | | 1198 - 1255 | 26326300 - 26326291 |  |
|  |  | |  |  |  |
| Exon 14 |  | | 1878 - 2386 | 26327916 - 26328485 |  |
| 14f | GAAGTTCAGGGAGGAGCTGG | | 1880 - 1899 | 26327979 - 26327998 |  |
| 14r | CAGGTATTCGTAGGGCCAGT | | 1942 - 1961 | 26328040 - 26328060 |  |
| probe set 14724105 |  | | 1970 - 1994 | 26328093 - 23628118 |  |
| ***IL13*** |  | | NM_001082594.1^2^ | NC_019462.1^3^ |  |
| Exon 1 |  | | 1 - 187 | 19264712 - 19254575 |  |
| 1f | CTCCATGGCGCTCTTCTTGA | | 51 - 70 | 19264705 - 19264868 |  |
| 1f | AGAGGAAGGCACAGGGTTTG | | 113 - 132 | 19264624 - 19264643 |  |
| probe set 14836731 |  | | 40 - 181 | 19264712 - 19264575 |  |
|  |  | |  |  |  |
| Exon 4 |  | | 404 - 1291 | 19262813 - 19261902 |  |
| 4f | GCCCAGGCACATTCCTTCTT | | 824 - 843 | 19262371 - 19262352 |  |
| 4r | GGCAGTAACAGTCCCTCCTAAC | | 897 - 918 | 19262277 - 19262298 |  |
| probe set 14836734 | |  | 453 - 1201 | 19262866 - 19262892 |  |
| ^1^ Accession number, mRNA sequence of Bos taurus *ALOX15*.  ^2^ Accession number, mRNA sequence of Ovis aries *IL13.*  ^3^ Accession number, OARv3.1 genome assembly | | | | | |
